# EMMPRIN Promotes Angiogenesis, Proliferation, Invasion and Resistance to Sunitinib in Renal Cell Carcinoma, and Its Level Predicts Patient Outcome

**DOI:** 10.1371/journal.pone.0074313

**Published:** 2013-09-20

**Authors:** Mototaka Sato, Yasutomo Nakai, Wataru Nakata, Takahiro Yoshida, Koji Hatano, Atsunari Kawashima, Kazutoshi Fujita, Motohide Uemura, Hitoshi Takayama, Norio Nonomura

**Affiliations:** The Department of Urology, Osaka University Graduate School of Medicine, Osaka, Japan; UCSF / VA Medical Center, United States of America

## Abstract

**Purpose:**

Extracellular matrix metalloproteinase inducer (EMMPRIN) has been reported to play crucial roles, including in angiogenesis, in several carcinomas. However, the correlation between EMMPRIN levels and angiogenesis expression profile has not been reported, and the role of EMMPRIN in renal cell carcinoma (RCC) is unclear. In the present study, we evaluated the association of EMMPRIN with angiogenesis, its value in prognosis, and its roles in RCC.

**Experimental Design:**

EMMPRIN expression was examined in 50 RCC patients treated with radical nephrectomy. Angiogenesis, proliferation, and invasion activity were evaluated using EMMPRIN knockdown RCC cell lines. The size of EMMPRIN-overexpressing xenografts was measured and the degree of angiogenesis was quantified. EMMPRIN expression was evaluated in RCC patients who received sunitinib therapy and in sunitinib-resistant cells. Further, the relation between EMMPRIN expression and sensitivity to sunitinib was examined.

**Results:**

EMMPRIN score was significantly associated with clinicopathological parameters in RCC patients, as well as being significantly correlated with microvessel area (MVA) in immature vessels and with prognosis. Down-regulation of EMMPRIN by siRNA led to decreased VEGF and bFGF expression, cell proliferation, and invasive potential. EMMPRIN over-expressing xenografts showed accelerated growth and MVA of immature vessels. EMMPRIN expression was significantly increased in patients who received sunitinib therapy as well as in sunitinib-resistant 786-O cells (786-suni). EMMPRIN-overexpressing RCC cells were resistant to sunitinib.

**Conclusion:**

Our findings indicate that high expression of EMMPRIN in RCC plays important roles in tumor progression and sunitinib resistance. Therefore, EMMPRIN could be a novel target for the treatment of RCC.

## Introduction

Renal cell carcinoma (RCC) accounts for 2–3% of all malignant tumors in adults, and clear cell renal cell carcinoma (ccRCC) is one of the most frequent RCC malignancies [1]. In most ccRCCs, inactivation of VHL tumor suppressor protein leads to activation of constitutive hypoxia-inducible transcription factor (HIF), an event thought to contribute to the regulation of vascular endothelial growth factor (VEGF) [2,3]. Thus, ccRCC is characterized by rich neovascularization and often a prominent vascular network around tumor cells. These tumors often metastasize via the vascular route, suggesting that tumor angiogenesis is important for ccRCC progression. In recent years, tyrosine kinase inhibitors (TKIs) have been developed to directly target angiogenesis signaling pathways in the progression of metastatic RCC (mRCC), and these novel targeted therapies are the standard of care recommended in most guidelines worldwide [4]. However, in most cases, these therapeutic options are offering no definitive cure. Even in cases that initially respond well to the targeted therapies, tumor regrowth occurs owing to chemoresistance. For these reasons, novel therapeutic targets are required to overcome resistance to TKIs.

Extracellular matrix metalloproteinase inducer (EMMPRIN), also known as cluster of differentiation (CD) 147, is a cell-surface glycoprotein that belongs to the immunoglobulin superfamily and it is encoded by a gene localized to 19p13.3 [5,6]. EMMPRIN has been implicated in many biological functions, including embryo implantation, spermatogenesis [7], and retinal development [8], and high EMMPRIN expression is observed in remodeling processes, such as inflammation, wound healing, and tumor progression [9-11]. Several studies of EMMPRIN in tumor tissues showed that increased expression is associated with clinically aggressive behavior and poor prognosis in a variety of human cancers [12-20], and Liang et al. [21] reported that EMMPRIN expression was significantly associated with prognosis in advanced RCC. The functions of EMMPRIN in tumors have been evaluated using many experimental methods. EMMPRIN stimulates cancer cells and peritumoral fibroblasts to secrete increased matrix metalloproteinases (MMPs), which are capable of degrading extracellular matrix (ECM) proteins, and EMMPRIN directly promotes tumor proliferation, invasion, and metastasis [16]. EMMPRIN has been reported to stimulate tumor angiogenesis via vascular endothelial cell growth factor (VEGF) [22,23], but the correlation of EMMPRIN with the degree of angiogenesis has not been reported, and the roles of EMMPRIN in RCC are unclear.

We previously investigated the degree of angiogenesis in RCC patients and found that microvessel area (MVA) of immature vessels was associated with tumor aggressiveness and prognosis in RCC patients [24]. In the present study, we investigated the correlation of EMMPRIN expression with MVA of immature vessels and with the prognosis of RCC patients and evaluated the role of EMMPRIN in determining the malignant potential of RCC cell lines. We further investigated the role of EMMPRIN in resistance to sunitinib, which is the first line TKI therapy in RCC.

## Materials and Methods

### Ethics statement

Written informed consent was obtained from all patients for the use of their tissue specimens, and the use of such specimens was approved by the Osaka University Hospital Institutional Review Board (Osaka, Japan). Animal experiments were approved by the Institutional Animal Care and Use Committee at Osaka University.

### Antibodies

Monoclonal anti-EMMPRIN, anti-CD34, anti-α-SMA, and anti-MCT1 antibodies were purchased from Abcam technology (Abcam Ltd, Cambridge, UK). HRP (horseradish peroxidase)-linked monoclonal anti-ERK, anti-p-ERK, anti-β-actin, and anti-rabbit IgG antibodies were purchased from Cell Signaling Technology (Danvers, MA, USA).

### Cell culture

786-O and Caki-1 human RCC cell lines were obtained from the American Type Culture Collection (Manassas, Virginia, USA). OUR-10 cell line was established in our laboratory [25]. 786-Suni is a sunitinib-resistant 786-O cell line that was established in our laboratory by exposing 786-O to sunitinib for 3 months. Sunitinib was purchased from Santa Cruz Biochemistry (Santa Cruz, CA, USA). All cell lines were grown in RPMI supplemented with 10% fetal bovine serum (FBS) and 1% penicillin-streptomycin solution (Invitrogen Corporation, Eugene, OR), and incubated at 37°C in a humidified atmosphere containing 5% CO2. The medium was changed twice per week.

### RNA transfection

siRNA for EMMPRIN and nontargeting control siRNA were purchased from QIAGEN. 786-O and OUR10 cells were transfected with the siRNA duplexes using HiPerFect Transfection Reagent (QIAGEN), according to the manufacturer’s protocol. Caki-1 cells were transfected with pEF-DEST51 vectors by electroporation following the manufacturer’s protocol. After transfection, Caki-1 cells were selected with blasticidin.

### MTS assay

All cell lines were seeded at 2 × 10^3^ cells/100 µL in 96-well plates and allowed to adhere overnight. After 24, 48, and 72 h incubation, 20 µL of CellTiter 96 AQueous One Solution Reagent (Promega, Wisconsin, USA) was added to each well, the trays were incubated for 1.5 h at 37°C, and the absorbance measured at 490 nm with a microplate reader.

### Invasion assay

The invasiveness of RCC cells was analyzed using a BD BioCoat Matrigel Invasion Chamber (BD, New Jersey, USA) (pore size, 8 µm). Serum-free RPMI containing 1 × 10^4^ cells in 500 µl was introduced into the upper compartment; the lower compartment contained 750 µl RPMI. After 48 h incubation at 37°C, cells that had penetrated the Matrigel were fixed with acetone and stained with H&E.

### Cytometric bead array (CBA)

All cell lines (2 × 10^4^) were cultured in 6-well plates. After 24 h the culture medium was replaced with 1 ml of fresh serum-free RPMI. After 3-days, conditioned medium was collected and whole-cell protein purified using RIPA Lysis Buffer (sc-24948; Santa Cruz Biotechnology, Inc). VEGF and bFGF proteins were measured in conditioned medium and in cell lysates using CBA, following the manufacturer’s protocol.

### Real-time reverse transcriptase PCR

Quantitative real-time PCR for EMMPRIN, bFGF and VEGF was performed with a Thermal Cycler Dice Real Time System TP800 (TaKaRa Bio, Shiga, Japan) using SYBR premix Ex TaqII (TaKaRa Bio). The QuantiTect Primer assay (QIAGEN) was used to detect expression of EMMPRIN, bFGF and VEGF, with β-actin as an internal control. PCR conditions were 50°C for 10 s and 95°C for 10 s followed by 32 cycles at 95°C for 15 s and 60°C for 1 min. EMMPRIN, bFGF, and VEGF mRNA levels were normalized to that of β-actin mRNA using the comparative *C*_T_ method. The primers used are shown in Table S1.

### Western blotting

Whole-cell protein was purified from each cell line with RIPA Lysis Buffer. After determining the protein concentration in the supernatant, 15 µg per well of protein was subjected to 10% PAGE, followed by western blotting with antibodies diluted as follows: EMMPRIN (1:1000), ERK (1:1000), p-ERK (1:1000), β-actin (1:1000), and MCT1 (1:500). Incubation was for 1 h at room temperature.

### Patients and samples

The study protocol was reviewed and approved by the appropriate institutional ethics committees. Tissue samples from 50 patients who underwent nephrectomy at Osaka University Hospital between 2002 and 2004 were enrolled in this study. None of the patients had received chemotherapy or radiotherapy. Two bilateral RCC patients and 5 patients who received neoadjuvant sunitinib therapy were evaluated. Histological diagnosis was established on standard hematoxylin and eosin-stained sections by 2 senior pathologists experienced in RCC diagnosis. Tumors were staged according to the 6^th^ AJCC TNM staging system and graded according to Fuhrman’s nuclear grading system. Survival data were available for all patients.

### Immunohistochemical staining and analysis

Formalin-fixed paraffin-embedded sections (5-µm thickness) were deparaffinized using xylene and alcohol and incubated with 0.3% H_2_O_2_ to block endogenous peroxidase activity. Before immunostaining, antigen was retrieved by immersing the sections in 10 mM citrate buffer (pH 6.0) and placed in the steam above boiling water for 20 min. Immunohistochemical stainings for EMMPRIN, CD34, α-SMA and MCT1 were performed using anti-EMMPRIN antibody (1:500), anti-CD34 antibody (1:500), anti-α-SMA antibody (1:500), and anti-MCT1 antibody (1:200) using the EnVision + Detection System (DAKO, Glostrup, Denmark) according to the manufacturer’s instructions. Primary antibodies were incubated for 60 min at room temperature and counterstained with hematoxylin. The levels of EMMPRIN staining were classified into 3 groups by scoring of positive cells: 2, strong; 1, moderate; and 0, weak. EMMPRIN score was then calculated by multiplying the staining intensity by the rate of positivity. Possible immunostaining scores ranged from 0 to 2. We evaluated 3 lesions for each tumor and calculated the mean score. Two independent investigators performed a blind evaluation of the immunostained slides.

### Animal models

Four-week-old male BALB/c nude mice were purchased from Japan SLC Inc. (Shizuoka, Japan) and maintained under standard conditions until the experiments were performed. Animals were fed a standard diet and water ad libitum. Animal welfare and experimental procedures were performed according to the NIH Guide for the Care and Use of Laboratory Animals. All protocols were approved by the Institutional Animal Care and Use Committee of Osaka University. Tumor cells were harvested with trypsin, resuspended in serum-free RPMI, and inoculated s.c. (1 × 10^7^/0.2 mL) into the left side of 3 nude mice per group. The size of the transplanted tumors was measured every 4 days and tumor volume was calculated using the formula V = 1/2 × (L × W^2^). Harvested tissues were fixed in 10% buffered formalin, embedded in paraffin, sectioned (5 µm), and then stained.

### Statistical analysis

Statistical analyses were performed using JMP9 (SAS Institute Inc). Results are presented as mean ± standard deviation (SD), and data were compared using the Wilcoxon test. Overall survival (OS) and progression-free survival (PFS) were calculated using the Kaplan-Meier method, and differences between groups were assessed by log rank tests. Multiple parametric groups were compared using ANOVA and then the Bonferroni post hoc test. Differences were considered statistically significant when p < 0.05.

## Results

### EMMPRIN score in RCC patients was associated with clinicopathological features, angiogenesis expression profile, and prognosis

**Figure 1:**
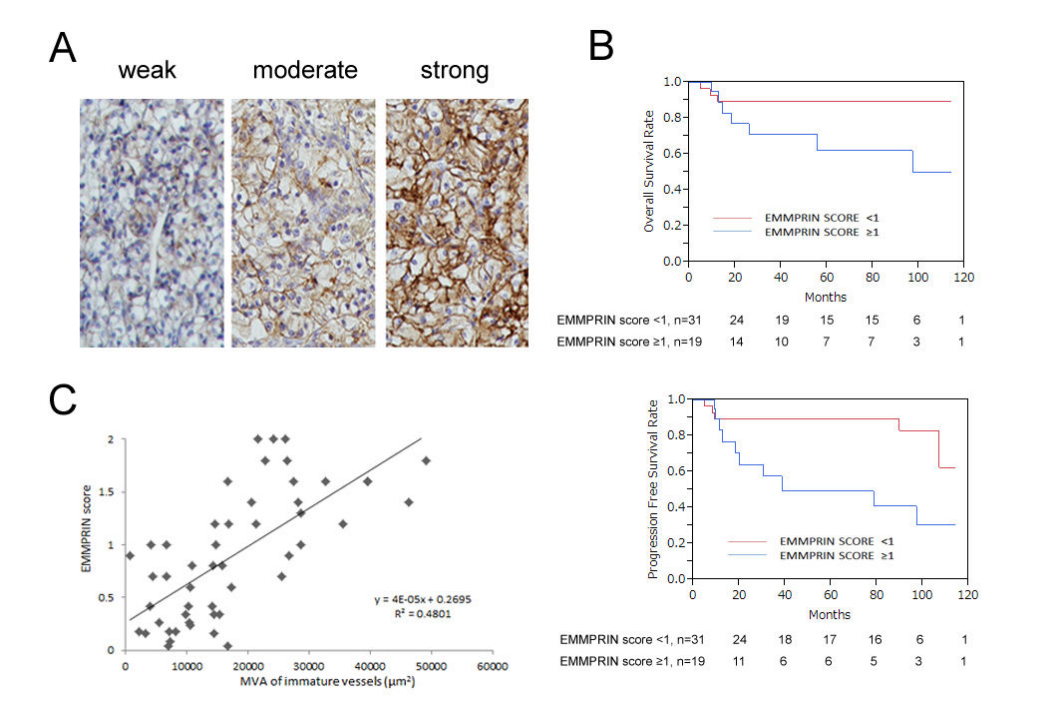
EMMPRIN was associated with the MVA of immature vessels and prognosis in RCC patients. (A) EMMPRIN staining pattern (left: weak, score 0; middle: moderate, score 1; right: strong, score 2) (× 200). (B) Effect of EMMPRIN expression level on overall survival (OS) and progression free survival (PFS) rates in RCC patients. (C) The correlation between EMMPRIN score and MVA of immature vessels in RCC patients.

EMMPRIN score was examined in 50 RCC patients through immunostaining. EMMPRIN was observed mainly on the cell surface. Representative examples of weak, moderate, and strong EMMPRIN staining are shown in Figure 1A. Staining intensity was weak in 14 samples (28%), moderate in 18 (36%), and strong in a further 18 cases (36%). In all cases, normal area next to the tumor area was also stained and EMMPRIN was not specifically stained in normal tissues. Table 1 shows the association between EMMPRIN scores and clinicopathological features of RCC. EMMPRIN score was significantly associated with pathological T stage, clinical M stage, AJCC stage, and Fuhrman Grade of RCC (p < 0.0001, 0.0003, 0.0001, and < 0.0001 respectively). OS (p = 0.0345) and PFS (p = 0.0066) were significantly shorter in patients with an EMMPRIN score ≥1 than in patients with an EMMPRIN score <1 (Figure 1B). We investigated the correlation between EMMPRIN score and the degree of angiogenesis. We have previously analyzed the ability of immature vessels to predict the prognosis of patients with RCC, reporting that the MVA of immature vessels may be a novel prognostic factor. The EMMPRIN score was significantly correlated with the MVA of immature vessels (R^2^ = 0.4801) (Figure 1C). These data demonstrate that EMMPRIN expression was associated with angiogenesis, clinical features, and prognosis in RCC patients.

**Table 1:** Clinicopathological characteristics and EMMPRIN score in 50 RCC patients who received radical nephrectomy.

Characteristics		Total (n = 50)	EMMPRIN score	p
Gender	Male	32	0.97 ± 0.58	0.3265
	Female	18	0.80 ± 0.64	
Age, years (Median)		27-82 (62)		
Pathological T stage	T1	32	0.62 ± 0.49	< 0.0001
	T2	8	1.32 ± 0.51	
	T3 / 4	10	1.48 ± 0.38	
Clinical M stage	M0	36	0.70 ± 0.52	0.0003
	M1	14	1.39 ± 0.51	
AJCC stage	I	29	0.57 ± 0.46	0.0001
	II	4	1.01 ± 0.51	
	III	3	1.37 ± 0.33	
	IV	14	1.41 ± 0.51	
Fuhrman grade	G1	28	0.55 ± 0.39	< 0.0001
	G2	17	1.34 ± 0.59	
	G3 / 4	5	1.40 ± 0.32	
Histological type	Clear cell	41	0.89 ± 0.63	0.695
	Non clear cell	9	0.94 ± 0.52	
Follow up, Month (Median)		1-114 (52)		

### Downregulation of EMMPRIN by siRNA decreased soluble VEGF and intracellular bFGF in RCC cell lines

**Figure 2:**
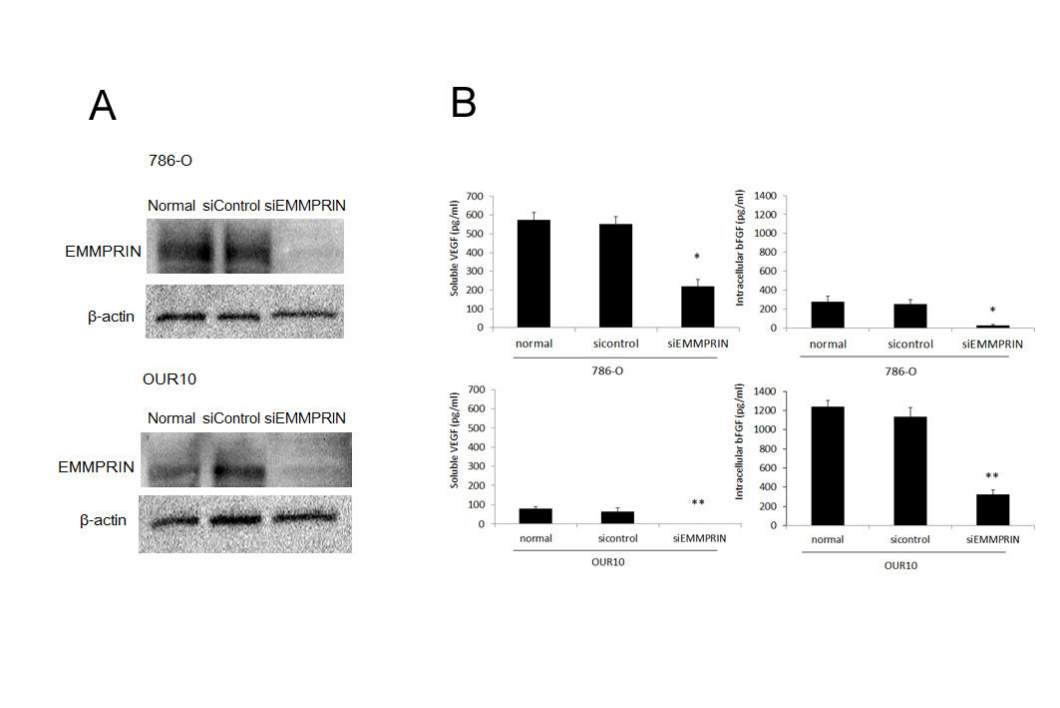
Transient siRNA knockdown of EMMPRIN decreased the expression of soluble VEGF and intracellular bFGF proteins. (A) Downregulation of EMMPRIN by siRNA in 786-O and OUR10 cells showed a remarkable decrease of EMMPRIN protein relative to normal or siControl 786-O cells. (B) CBA showed siRNA EMMPRIN decreased soluble VEGF protein expression and intracellular bFGF protein expression in RCC cells for 786-O compared with a 786-O siControl, and for OUR10 compared with an OUR10 siControl. *p: < 0.05.

Vascular endothelial growth factor (VEGF) and basic fibroblast growth factor (bFGF) are key factors in angiogenesis; hence, we evaluated the effect of silencing EMMPRIN on VEGF and bFGF expression. EMMPRIN was down-regulated in 2 RCC cell lines, 786-O and OUR10, and the effects of this down-regulation were evaluated using western blotting. Down-regulation of EMMPRIN by siRNA in 786-O and OUR10 was confirmed (Fig. 2A). Using CBA, we found that VEGF protein in RCC cell lines was detected mainly in the conditioned medium, not in cell-lysates; conversely, bFGF protein was detected mainly in cell-lysates, not in the conditioned medium, and total VEGF expression was higher in 786-O cells than in OUR-10 cells, whereas bFGF was more highly expressed in OUR10 (Figure S1). siRNA-mediated knockdown of EMMPRIN significantly decreased soluble VEGF and intracellular bFGF in both 786-O cells and OUR10 cells (p < 0.05 in all cases) (Figure 2B).

### Down-regulation of EMMPRIN significantly inhibited the proliferation and invasion of RCC cells

**Figure 3:**
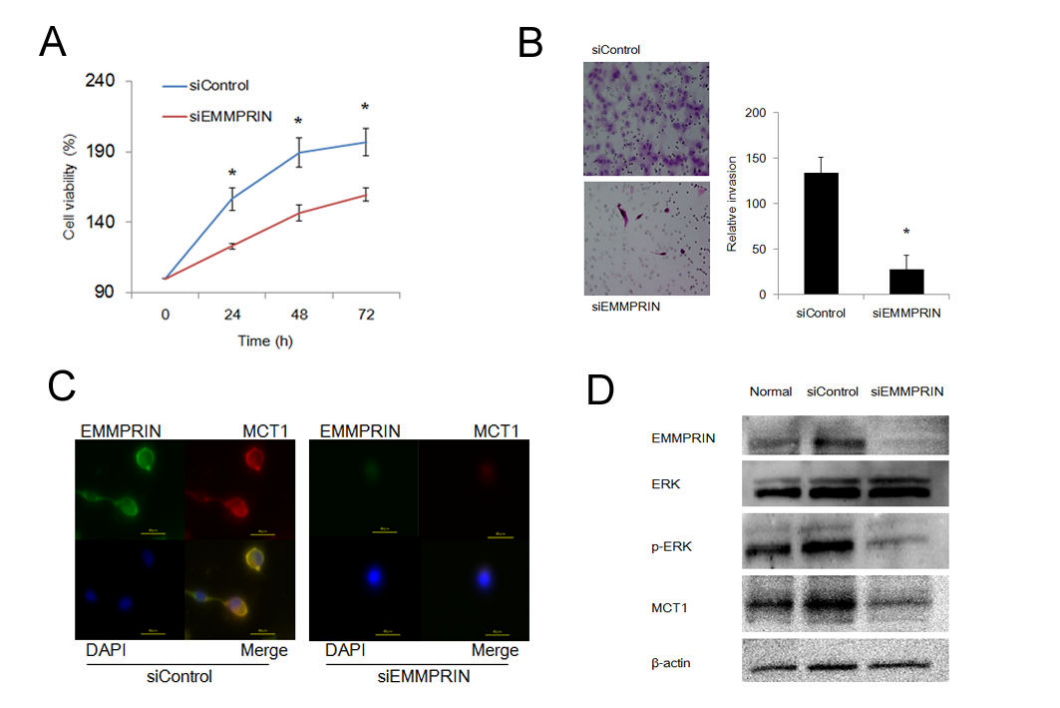
EMMPRIN siRNA significantly decreased the proliferation and invasion activities of 786-O. (A) MTS assay (at 24, 48, and 72 h) was performed after EMMPRIN knockdown in 786-O cells. EMMPRIN siRNA significantly inhibited the proliferation of 786-O cells. *: p < 0.05. (B) (Left) Representative images of invasive 786-O cells transfected with control and EMMPRIN siRNA in Matrigel invasion assays (× 200) (Right). Quantification of invaded cells. EMMPRIN siRNA significantly inhibited the invasiveness of 786-O cells. (C) 786-O cells were co-labeled with anti-EMMPRIN and anti-MCT1 antibodies (× 800). 786-O cells were co-localized on the membrane of 786-O cells. The co-localized expression of EMMPRIN and MCT1 was abolished by EMMPRIN siRNA. (D) Western blotting of MCT1 p-ERK protein expression. Expression was decreased by EMMPRIN siRNA in 786-O cells.

We examined the effects of EMMPRIN silencing on cell proliferation and invasion in these cell lines. As shown in Figure 3A, proliferation of siRNA-EMMPRIN 786-O cells was significantly attenuated (p < 0.05) compared with that of siRNA-control 786-O cells. Invasion assay using a Matrigel Invasion Chamber showed that siRNA-EMMPRIN 786-O cells significantly inhibited invasion activity (p = 0.0003) compared with the siRNA-Control 786-O cells (Figure 3B). Similar results were obtained with OUR10 cells (Figure S2A, B).

### EMMPRIN and MCT1 co-localized, and this was inhibited by EMMPRIN silencing

Rapid tumor progression under hypoxic conditions is mediated by glycolysis, and increased glycolysis leads to excessive intracellular lactic acid production. Lactic acid is excreted from cells through the monocarboxylate transporter (MCT). To investigate the association between EMMPRIN and MCT1, 786-O cells were co-labeled with anti-EMMPRIN and anti-MCT1 antibodies and observed under an immunofluorescence confocal microscope. EMMPRIN and MCT1 were co-localized in the plasma membrane, and when EMMPRIN was silenced, we observed a significant decrease in this co-localized expression (Figure 3C). The results of western blotting also showed that whole-cell MCT1 protein expression was decreased by EMMPRIN siRNA in 786-O cells (Figure 3D). OUR-10 cells also showed these effects (Figure S2C, D).

### EMMPRIN silencing decreased ERK phosphorylation

Evaluation of several signaling pathways has demonstrated a link between EMMPRIN and the MAP cascade [26]. In both 786-O and OUR10 cell lines, western blotting showed EMMPRIN silencing decreased ERK phosphorylation in whole cell lysates (Figure 3D, Figure S2D).

### Effects of EMMPRIN overexpression on tumor growth and angiogenesis *in vivo*

**Figure 4:**
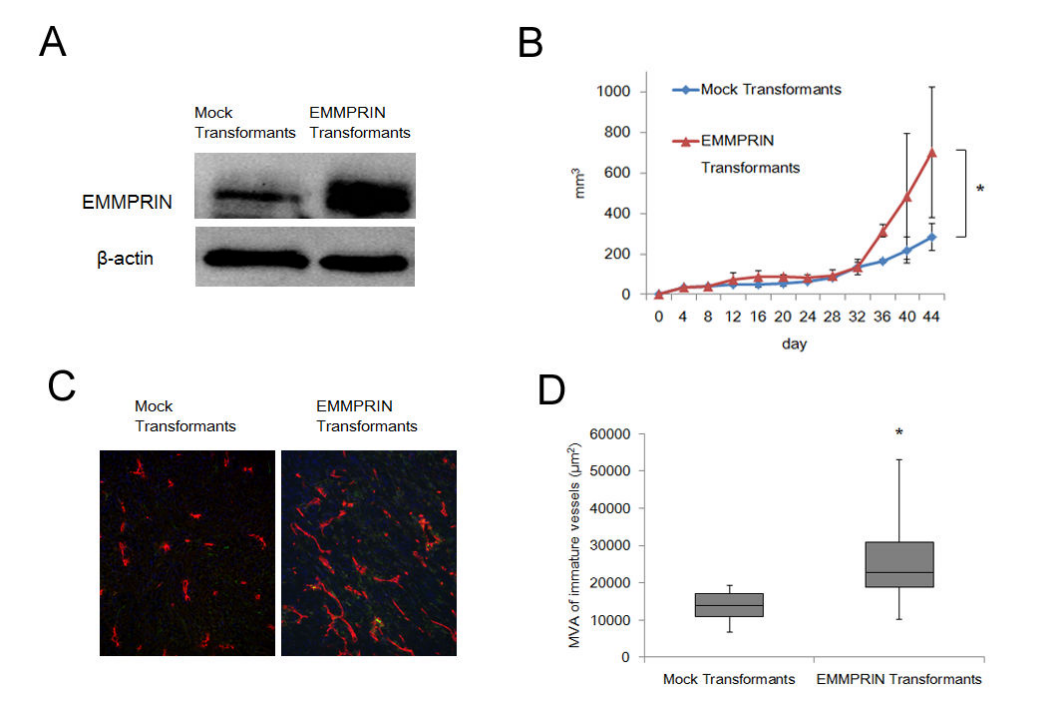
Effects of EMMPRIN overexpression on tumor growth and angiogenesis *in vivo*. (A) Western blotting of whole cell lysates from stably transfected Caki-1 cells showed remarkable EMMPRIN protein overexpression. (B) The growth curves of transplanted tumors in nude mice. Caki-1 cells were injected s.c. into the left axillary fossa and tumor volumes were measured. (C) Immunohistochemical analysis of angiogenesis in transplanted tumors (Red: CD34) (Left). Caki-1 mock transformants (×200); (Right) Caki-1 EMMPRIN transformants (×200). (D) MVA of immature vessels in transfected tumors was measured. Caki-1 EMMPRIN transformants had a significantly higher MVA of immature vessels than Caki-1 mock transformants (p = 0.0118).

EMMPRIN was stably overexpressed in Caki-1, an RCC cell line with relatively low expression of EMMPRIN (Figure 4A). We inoculated this cell line in immunodeficient nude mice to determine the effect of EMMPRIN overexpression on tumorigenicity and angiogenic potential. The volume and MVA of immature vessels in xenografts were calculated. After 44 days, the tumor volume in mice inoculated with Caki-1 EMMPRIN transformants was 248% greater than in mice inoculated with Caki-1 mock transformants (p < 0.05) (Figure 4B). The MVA of immature vessels in EMMPRIN transformants was 26997 ± 13683 µm^2^, whereas it was 1356 ± 4450 µm^2^ in mock tumors. This difference is statistically significant (p < 0.0118) (Figure 4C, D).

### Influence of EMMPRIN on sunitinib resistance in RCC

**Figure 5:**
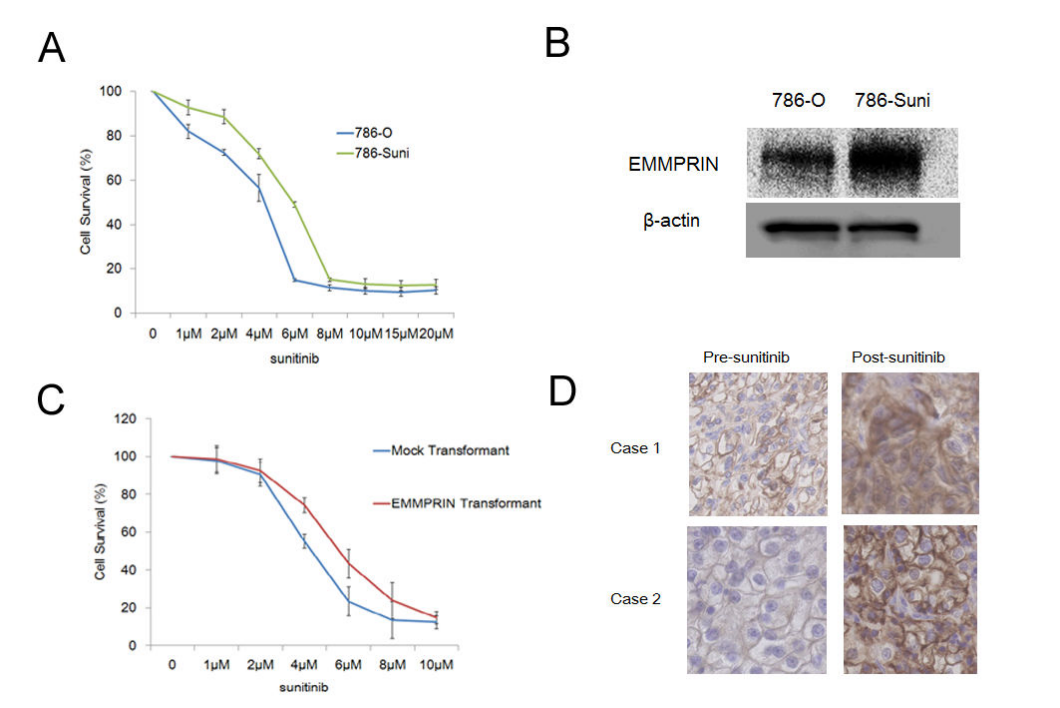
EMMPRIN levels in sunitinib-resistant RCC. (A) Characterization of sunitinib resistant 786-O. Cell survival of 786-O and 786-Suni cells exposed to sunitinib for 72 h. (B) EMMPRIN protein expression in 786-O and 786-Suni cells. (C) Anti-proliferative effects of sunitinib on Caki-1 mock transformants and Caki-1 EMMPRIN transformants. (D) EMMPRIN staining in 2 bilateral RCC patients (left: pre sunitinib treatment, right: post sunitinib treatment) (×200).

TKIs and mTOR inhibitors targeting angiogenesis have been clinically used in the treatment of RCC, but the effect of these agents is limited and resistance becomes a problem. We investigated the role of EMMPRIN in sunitinib resistance. Continuous exposure of 786-O cells to sunitinib led to the development of 786-Suni cells. The half-maximum inhibitory concentration (IC_50_) of sunitinib increased from 4.9 µM in parental 786-O cells to 6.8 µM in sunitinib-resistant 786-Suni cells (Figure 5A). 786-Suni cells displayed increased EMMPRIN protein expression compared with parental cells (Figure 5B). We evaluated the anti-proliferative effects of sunitinib in Caki-1 mock transformants and Caki-1 EMMPRIN transformants. The IC_50_ of sunitinib was increased from 4.7 µM in Caki-1 mock transformant cells to 6.0 µM in Caki-1 EMMPRIN transformant cells (Figure 5C). We examined EMMPRIN expression in 2 RCC patients who had bilateral RCC. These patients had first undergone nephrectomy for a unilateral tumor, and after sunitinib treatment, a contralateral tumor was partially nephrectomized. Figure 5D shows elevated expression of EMMPRIN in the 2 patients after sunitinib treatment. Next, we evaluated the EMMPRIN score of 5 patients who had neoadjuvant sunitinib treatment. These patients had a high EMMPRIN score (Table S2). These data may suggest that the use of sunitinib leads to EMMPRIN expression.

## Discussion

In the present study, we showed that the expression level of EMMPRIN in RCC patients was associated with clinicopathological features, degree of angiogenesis, and prognosis. Silencing EMMPRIN in RCC cells significantly decreased VEGF, bFGF, and MCT1 and activated ERK expression, proliferation, and invasion in RCC cell lines. *In vivo*, EMMPRIN overexpression accelerated tumor growth and angiogenesis activity. Additionally, EMMPRIN overexpression leads to sunitinib resistance, and treatment with sunitinib effectively selects RCC cells that overproduce EMMPRIN.

Angiogenesis is critical for the progression and metastasis of malignant tumors [27], and the degree of angiogenesis has been reported to predict progression and survival for several carcinoma types [28-31]. RCC is the most common kidney cancer and is one of the most vascularized solid tumors. In recent years, TKIs and mTOR inhibitors targeting angiogenesis have been used clinically. However, these therapeutic options are only temporary, and inevitably, tumor progression will occur in most patients owing to chemoresistance. Therefore, there is an urgent need to develop novel systemic agents.

EMMPRIN, originally identified as a cell-surface protein of the immunoglobulin superfamily (27, 28), is highly expressed on the surface of various tumor cells compared with their normal counterparts (12, 13, 29) and is involved in tumor progression (30, 31). Overexpression of EMMPRIN in cancer tissues has been reported to be associated with poor prognosis in cancer patients [32,33]. In the present study, as expected, we demonstrated that EMMPRIN score was positively associated with tumor grade, T stage, and AJCC stage and that it was significantly higher in tumors with metastasis than in those without metastasis. PFS and OS were significantly shorter in patients with a high EMMPRIN score than in patients with a low EMMPRIN score.

The degree of angiogenesis has been quantified using several factors including MVA, microvessel density (MVD), expression of angiogenic molecules within the tumor, and presence of angiogenic receptors within tumor tissue [34-38]{Liotta, 1974, Quantitative relationships of intravascular tumor cells’, tumor vessels’, and pulmonary metastases following tumor implantation}. We previously analyzed the ability of immature vessels to predict the prognosis of patients with RCC, and the MVA of immature vessels was significantly associated with prognosis. In the present study, we demonstrated a positive correlation between EMMPRIN score and the MVA of immature vessels in RCC patients. To the best of our knowledge, this is the first evidence that EMMPRIN can affect tumor angiogenesis.

Dozens of angiogenic factors and cytokines are overexpressed in many tumor types [39]. Among these angiogenic factors, the VEGF family has been a central focus in tumor angiogenesis research, and agents that selectively target the VEGF/VEGFR system, such as sunitinib, sorafenib, and bevacizumab, have shown promising activity in clinical trials and have been approved for use in selected cancer indications [40]. However, angiogenesis depends on multiple factors, and when the activity of one angiogenic factor such as VEGF is suppressed, the expression of other angiogenic factors appears to emerge [41]. bFGF is one of the major angiogenesis factors in human cancer, and experimental evidence indicates that drug resistance to VEGF blockade may lead to reactivation of angiogenesis triggered by the bFGF/bFGFR system. Upregulation of this system represents one of the mechanisms for overcoming anti-VEGF therapy in cancer treatment [42,43].

EMMPRIN was reported to promote angiogenesis mainly by elevating the expression of VEGF [23]. In the present study, we evaluated both VEGF and bFGF expressions in RCC cell lines. Interestingly, VEGF and bFGF were expressed differently in different cell lines, and the levels of both were decreased by the silencing of EMMPRIN. EMMPRIN expression correlated with the MVA of immature vessels in clinical and xenograft models, suggesting EMMPRIN as a target for new, more effective antiangiogenic drugs. The effect of EMMPRIN siRNA on VEGF and bFGF gene expression was analyzed at the mRNA level (Figure S3). bFGF mRNA level was reduced by 36% in 786-O cells and by 43% in OUR10 cells (p < 0.05 for both lines), but the VEGF mRNA level was not reduced by EMMPRIN siRNA, indicating that EMMPRIN acts on VEGF translation or secretion.

The well-known function of EMMPRIN is to induce the secretion of various MMPs, which degrade the extracellular matrix and promote the invasion and metastasis of cancer cells [5], and EMMPRIN was also reported to directly promote proliferative and invasive potential in malignant melanoma [44]. Indeed, in the present study, EMMPRIN-targeting siRNA inhibits proliferation and invasion by RCC cell lines. We evaluated variations in glycolysis and the downstream MAP signaling pathway as possible causes of this phenomenon. MCTs are responsible for H^+^-linked transport of monocarboxylates across the cell membrane. It was recently demonstrated that proper plasma membrane localization and activity of MCTs require the presence of EMMPRIN as a chaperone and that MCT-1 acts as chaperone for EMMPRIN [45]. Indeed, this study showed that silencing of EMMPRIN decreased MCT1 expression and abolished the co-localization of EMMPRIN and MCT1. EMMPRIN might promote tumor aggressiveness via the glycolysis pathway.

ERK signaling can regulate disparate processes such as proliferation, differentiation, survival, migration, angiogenesis, and chromatin remodeling in a cell type-dependent manner. In human hepatoma cells, activation of the ERK pathway reportedly leads to survival or cell proliferation [46]. Our RNA interference studies indicate that tumor-associated EMMPRIN promoted the activated ERK in RCC cell lines. This also indicates the involvement of EMMPRIN in tumor progression.

Multidrug resistance is an important cause of treatment failure and mortality. EMMPRIN is reported to be involved in multidrug resistance of cancer cells via hyaluronan-mediated activation of ErbB2 signaling and cell survival pathway activities [47,48]. Bo Wang et al. reported that RNAi-mediated silencing of EMMPRIN promotes tumor sensitivity to cisplatin in a human gastric cancer cell line [49]. Recently, sunitinib was approved for use in mRCC, and the use of sunitinib was demonstrated to have survival benefits [50]. However, most patients treated with sunitinib eventually develop resistance and experience disease progression. In the present study, we investigated whether EMMPRIN affects resistance to sunitinib in RCC. Clinical data showed that after sunitinib therapy, RCC had higher EMMPRIN score, and sunitinib-resistant RCC cells also expressed more EMMPRIN. EMMPRIN overexpressing cells were more resistant to sunitinib. To the best of our knowledge, this is the first evidence that EMMPRIN may be involved in sunitinib resistance.

EMMPRIN is highly expressed in human RCC cells, and it plays an important role in their angiogenesis and aggressiveness both *in vitro* and *in vivo*. Further studies are warranted to validate that targeting EMMPRIN in RCC inhibits tumor angiogenesis, progression, and resistance to TKIs and mTOR inhibitors.

## Conclusions

We have shown that the expression level of EMMPRIN in RCC is associated with malignancy, angiogenesis, and prognosis. Silencing EMMPRIN significantly decreased VEGF and bFGF expression, MCT1 colocalization, expression of activated ERK, proliferation, and invasiveness in RCC cells. EMMPRIN may also lead to the development of resistance to sunitinib in RCC. Our findings indicate that high expression of EMMPRIN in RCC plays an important role in tumor progression. These data indicate that EMMPRIN may be a novel target for the treatment of RCC.

## Supporting Information

Figure S1 VEGF and bFGF protein expression in conditioned medium and cell-lysate for cultured 786-O and OUR10 cells (*: p < 0.05). (A, B) VEGF protein was expressed more in conditioned medium than in cell-lysates in 786-O and OUR10 cells (p < 0.05 and p < 0.05, respectively). (C, D) bFGF protein was expressed more in cell-lysates than in conditioned medium in 786-O and OUR10 cells (p < 0.05 and p < 0.05, respectively).
(TIF)

Figure S2 **EMMPRIN siRNA significantly inhibited proliferation and invasion activities of OUR10.** (A) MTS assay (at 24, 48, and 72 h) was performed after EMMPRIN knockdown in OUR10 cells. EMMPRIN siRNA significantly inhibited the proliferation of OUR10 cells. *: p < 0.05. (B) (Left) Representative images of invasive OUR10 cells transfected with control and EMMPRIN siRNA in Matrigel invasion assays (× 200) (Right). Quantification of invaded cells. EMMPRIN siRNA significantly inhibited the invasiveness of OUR10 cells. (C) OUR10 cells were co-labeled with anti-EMMPRIN and anti-MCT1 antibodies (×800). OUR10 cells were co-localized on the membrane of OUR10 cells. The co-localized expression of EMMPRIN and MCT1 was abolished by EMMPRIN siRNA. (D) Western blotting of MCT1 p-ERK protein expression. Expression was decreased by EMMPRIN siRNA in OUR10 cells.
(TIF)

Figure S3 **RT-PCR showed EMMPRIN siRNA decreased soluble bFGF gene expression in 786-O and OUR10 RCC cells (p < 0.05 and p < 0.05, respectively).** VEGF gene expression was not decreased in these cells.
(TIF)

Table S1 **Sequences of the primers used in this study.**
(DOC)

Table S2 **Clinicopathological parameters and EMMPRIN score for RCC patients who had neoadjuvant sunitinib treatment.**
(DOC)
