# Supplementary figures and images for: EMMPRIN Promotes Angiogenesis, Proliferation, Invasion and Resistance to Sunitinib in Renal Cell Carcinoma, and Its Level Predicts Patient Outcome
Source: PLoS One. 2013 Sep 20;8(9):e74313. doi: 10.1371/journal.pone.0074313 (PMC3779201; doi:10.1371/journal.pone.0074313)

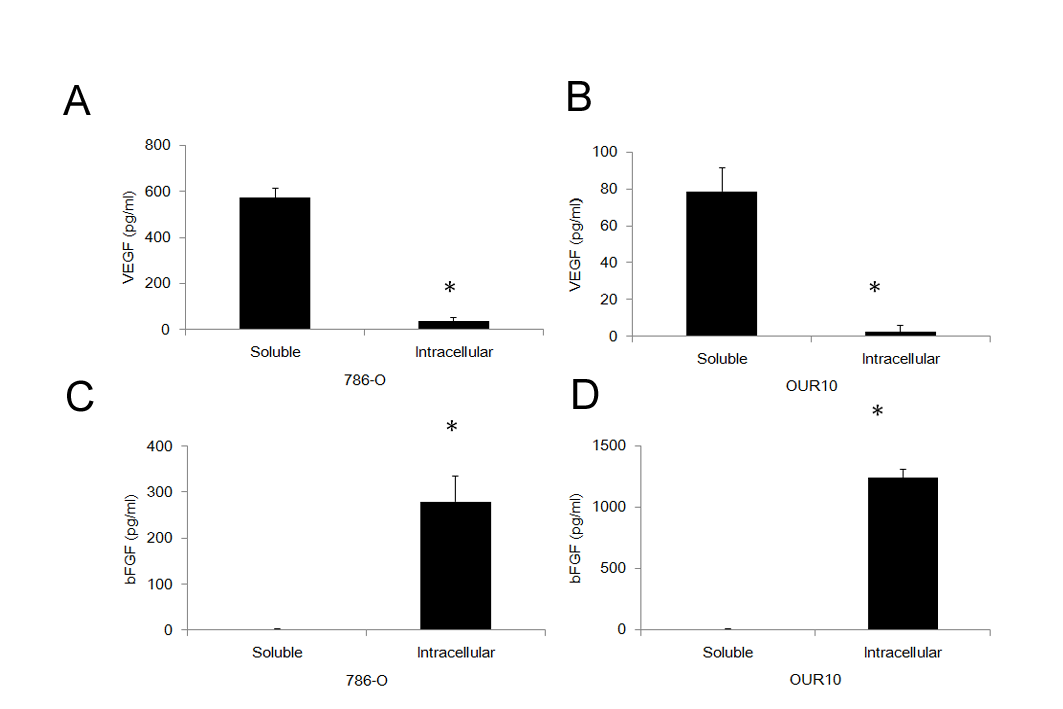

Supplement: Figure S1 — VEGF and bFGF protein expression in conditioned medium and cell-lysate for cultured 786-O and OUR10 cells (*: p < 0.05). (A, B) VEGF protein was expressed more in conditioned medium than in cell-lysates in 786-O and OUR10 cells (p < 0.05 and p < 0.05, respectively). (C, D) bFGF protein was expressed more in cell-lysates than in conditioned medium in 786-O and OUR10 cells (p < 0.05 and p < 0.05, respectively). (TIF) [file pone.0074313.s001.tif]

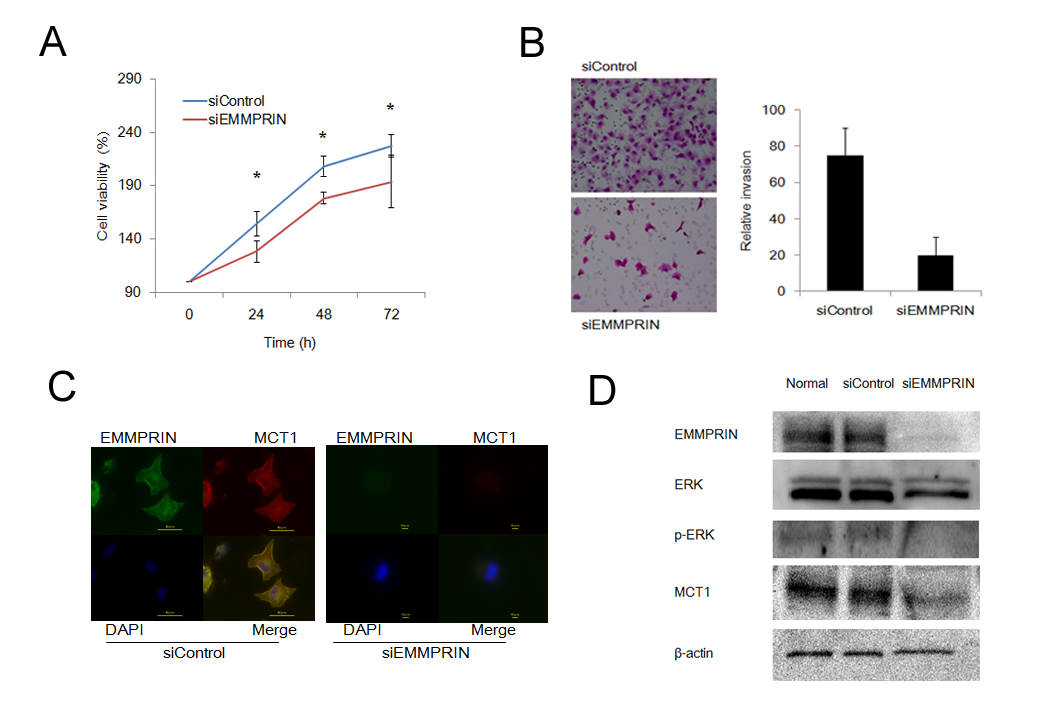

Supplement: Figure S2 — EMMPRIN siRNA significantly inhibited proliferation and invasion activities of OUR10. (A) MTS assay (at 24, 48, and 72 h) was performed after EMMPRIN knockdown in OUR10 cells. EMMPRIN siRNA significantly inhibited the proliferation of OUR10 cells. *: p < 0.05. (B) (Left) Representative images of invasive OUR10 cells transfected with control and EMMPRIN siRNA in Matrigel invasion assays (× 200) (Right). Quantification of invaded cells. EMMPRIN siRNA significantly inhibited the invasiveness of OUR10 cells. (C) OUR10 cells were co-labeled with anti-EMMPRIN and anti-MCT1 antibodies (×800). OUR10 cells were co-localized on the membrane of OUR10 cells. The co-localized expression of EMMPRIN and MCT1 was abolished by EMMPRIN siRNA. (D) Western blotting of MCT1 p-ERK protein expression. Expression was decreased by EMMPRIN siRNA in OUR10 cells. (TIF) [file pone.0074313.s002.tif]

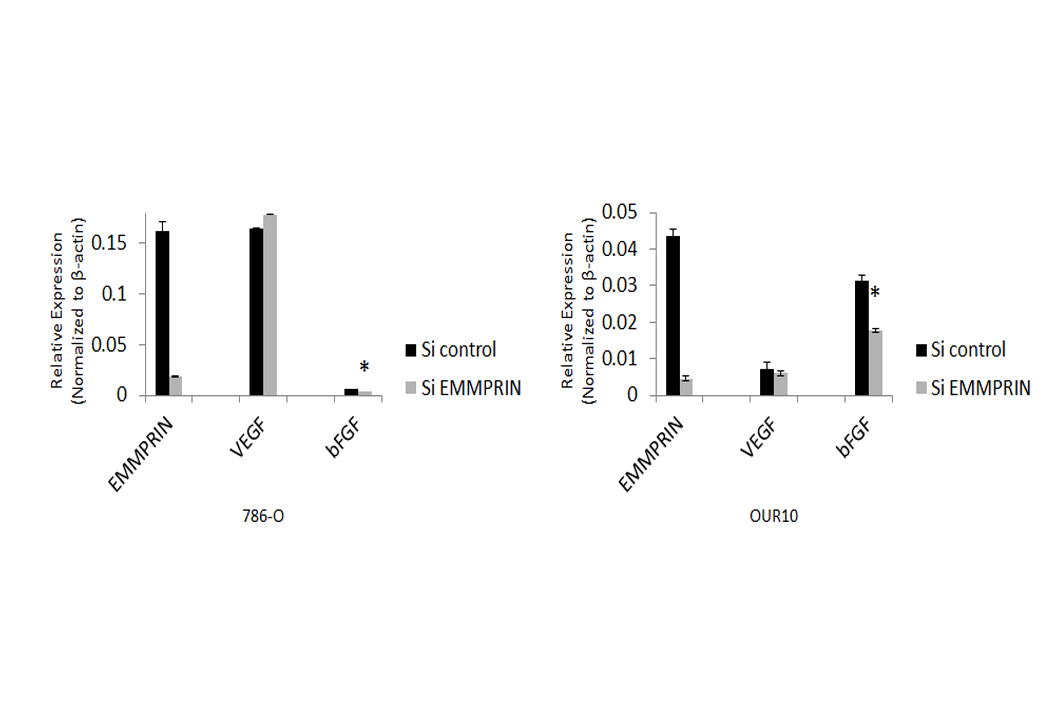

Supplement: Figure S3 — RT-PCR showed EMMPRIN siRNA decreased soluble bFGF gene expression in 786-O and OUR10 RCC cells (p < 0.05 and p < 0.05, respectively). VEGF gene expression was not decreased in these cells. (TIF) [file pone.0074313.s003.tif]
