# Supplementary material for: EMMPRIN Promotes Angiogenesis, Proliferation, Invasion and Resistance to Sunitinib in Renal Cell Carcinoma, and Its Level Predicts Patient Outcome
Source: PLoS One. 2013 Sep 20;8(9):e74313. doi: 10.1371/journal.pone.0074313 (PMC3779201; doi:10.1371/journal.pone.0074313)
Supplement: Table S1 — Sequences of the primers used in this study. (DOC) [file pone.0074313.s004.doc]

Table S1. Sequences of the primers used in this study.

| Gene name | Forward primer (5'-3') | Reserve primer (5'-3') |
| --- | --- | --- |
| β-actin | CTAAGTCATAGTCCGCCTAGAAGCA | TGGCACCCAGCACAATGAA |
| EMMPRIN | CAGCGGTTGGAGGTTGT | TTTGAGGGTGGAGGTGG |
| VEGF | AGCTACTGCCATCCAATCGA | GGTGAGGTTTGATCCGCATA |
| bFGF | CTGTGCTAACCGTTACCTGGCTATG | CCAGTTCGTTTCAGTGCCACA |
| Sequences of the primers used for this study | |  |
