# Supplementary material for: EMMPRIN Promotes Angiogenesis, Proliferation, Invasion and Resistance to Sunitinib in Renal Cell Carcinoma, and Its Level Predicts Patient Outcome
Source: PLoS One. 2013 Sep 20;8(9):e74313. doi: 10.1371/journal.pone.0074313 (PMC3779201; doi:10.1371/journal.pone.0074313)
Supplement: Table S2 — Clinicopathological parameters and EMMPRIN score for RCC patients who had neoadjuvant sunitinib treatment. (DOC) [file pone.0074313.s005.doc]

Table S2. Clinicopathological parameters and EMMPRIN score for RCC patients who had neoadjuvant sunitinib treatment.

| Case | Neoaduvant therapy | effect | Pathological T stage | Fuhrman Grade | EMMPRIN score (0-2) |
| --- | --- | --- | --- | --- | --- |
| 1 | Sunitinib 2course | SD | pT1a | 1 | 1.4 |
| 2 | Sunitinib 1course | PD | pT1b | 1 | 2 |
| 3 | Sunitinib 2course | SD | pT2 | 2 | 2 |
| 4 | Sunitinib 2course | SD | pT3a | 2 | 2 |
| 5 | Sunitinib 1course | PD | pT4 | 3 | 1.67 |
|  | SD: Stable Disease |  | PD: Progressive Disease |  |  |
